# Molecular and cellular mechanisms leading to catatonia: an integrative approach from clinical and preclinical evidence

**DOI:** 10.3389/fnmol.2022.993671

**Published:** 2022-09-29

**Authors:** Daniel Felipe Ariza-Salamanca, María Gabriela Corrales-Hernández, María José Pachón-Londoño, Isabella Hernández-Duarte

**Affiliations:** ^1^Medical and Health Sciences Education Research Group, School of Medicine and Health Sciences, Universidad del Rosario, Bogotá, Colombia; ^2^Pharmacology Unit, Department of Biomedical Sciences, School of Medicine and Health Sciences, Universidad del Rosario, Bogotá, Colombia

**Keywords:** catatonia, mechanism, neuroinflammation, oxidative stress, NMDA

## Abstract

This review aims to describe the clinical spectrum of catatonia, in order to carefully assess the involvement of astrocytes, neurons, oligodendrocytes, and microglia, and articulate the available preclinical and clinical evidence to achieve a translational understanding of the cellular and molecular mechanisms behind this disorder. Catatonia is highly common in psychiatric and acutely ill patients, with prevalence ranging from 7.6% to 38%. It is usually present in different psychiatric conditions such as mood and psychotic disorders; it is also a consequence of folate deficiency, autoimmunity, paraneoplastic disorders, and even autistic spectrum disorders. Few therapeutic options are available due to its complexity and poorly understood physiopathology. We briefly revisit the traditional treatments used in catatonia, such as antipsychotics, electroconvulsive therapy, and benzodiazepines, before assessing novel therapeutics which aim to modulate molecular pathways through different mechanisms, including NMDA antagonism and its allosteric modulation, and anti-inflammatory drugs to modulate microglia reaction and mitigate oxidative stress, such as lithium, vitamin B12, and NMDAr positive allosteric modulators.

## Introduction

Catatonia is a complex neuropsychiatric syndrome and since its recognition, it was described as a symptom or consequence of schizophrenia (SCZ). A great body of neuroscientific evidence has thrown up interesting hypotheses around cellular and circuitry defects leading to catatonia. The clinical spectrum of catatonia is wide, mostly involving affective and motor control-related encephalic areas (Walther and Strik, 2016). Its diverse clinical spectrum, which will be later discussed, makes an accurate diagnosis difficult. During the past three decades, therapeutics have remained relatively constant, with benzodiazepines being the first line of treatment; however, they are not always effective. The wide application of electroconvulsive therapy (ECT) in psychiatric disorders has shown great efficacy, including catatonia (Dhossche and Withane, 2019; Kellner et al., 2020). Nonetheless, a definitive, specific treatment is still to be elucidated.

The epidemiological data surrounding catatonia is variable and depends on the sample studied and the methods used to diagnose it (Stuivenga and Morrens, 2014). The establishment of catatonic symptomatology is frequently related to other psychiatric conditions and acutely ill patients. The most frequent association was first noticed with schizophrenia, with a prevalence of up to 50% depending on the study (Deister and Marneros, 1994; Bräunig et al., 1998; Ungvari et al., 2005); nonetheless, current data establishes, varying depending on the population, a coexistence of schizophrenia/catatonia between 9.8% and 20% (Stuivenga and Morrens, 2014; Rasmussen et al., 2016; Solmi et al., 2018). A systematic review presented by Solmi et al. (2018) studied the epidemiological heterogeneity regarding the coexistence between catatonia and schizophrenia, more than half of the 74 studies included presented a European population whereas a study performed by Zingela et al. (2022) reported a coexisting prevalence of 47.7% among South African population. Said statistical difference not only exemplifies changes in diagnostic tools and criteria throughout the years, but it also leaves a significant number of catatonic patients in whom no clear etiology is known thus encouraging further studies on the subject. Interestingly, a stronger association has been elucidated between catatonia and affective disorders, with the prevalence of up to 20% to 40% in depression and mania respectively (Bräunig et al., 1998; Solmi et al., 2018).

It is worth bearing in mind that catatonia can emerge with no prior psychiatric comorbidity or in the presence of medical and neurological illness, with an estimated prevalence of 20.6% (Solmi et al., 2018; Connell et al., 2021). As for autism, an early-onset neurodevelopmental disorder, its similar and sometimes overlapping clinical manifestations have led clinicians and investigators to study a possible correlation between these two entities (Wing and Shah, 2000). Many cases have clearly related autoimmunity to the onset of catatonia: anti-N-methyl-D-aspartate receptor (NMDAR) encephalitis, autoimmune thyroid disorders, multiple sclerosis, and systemic lupus erythematosus (Rogers et al., 2019; Durns et al., 2020). Other authors pinpointed alterations of the limbic system like lower volumes of the amygdala and the hypothalamus as the arise of the affective component of catatonia (Fritze et al., 2022). Furthermore, a widely known hypothesis is anomalies in dopamine and GABAergic circuitries result in deficits of top-down modulation (Northoff, 2002). This puzzle has forced researchers to formulate a comprehensive approach to the pathophysiology of catatonia beyond an alteration in neuron circuitry.

The mechanisms underlying catatonia onset have been broadly studied; however, there is no consensus on how or why this syndrome occurs. Current explanations involve dysfunctions in encephalic areas such as the supplementary motor area (SMA), orbitofrontal cortex (OFC), prefrontal cortex (PFC; Haroche et al., 2020), and gamma-Aminobutyric acid-A (GABA-A) and N-Methyl-D-Aspartate (NMDA) receptor dysfunction has been proposed as well (Moussa et al., 2019; Samra et al., 2020). Additionally, evidence of the role of glia in neuropsychiatric disorders is growing (González-Reyes et al., 2017; Vargas-Sánchez et al., 2018; Baracaldo-Santamaría et al., 2022), and when assessing preclinical evidence around catatonia, animal models inducing symptoms resembling catatonia have focused on the interactions between neurons and glia; moreover, they have tried to modulate glial activation with encouraging results (Hagemeyer et al., 2012). Therefore, it is crucial to elaborate on an integrative approach that includes the complex interactions between neurons, microglia, oligodendrocytes, and astrocytes.

In this review, we carefully assess clinical tools to diagnose catatonia and the preclinical evidence in experimental catatonic models, deeply emphasizing processes that alter microglia, astrocytes, and oligodendrocytes. We explore clinical evidence from an extensive analysis of reported cases and functional images in catatonic patients, as well as the best literature available for similar diseases, in order to help us frame the understanding of this complex syndrome. Furthermore, regarding the cellular and circuitry pathways, we will review and propose current and novel therapeutics that might help to treat catatonia.

## Clinical Spectrum and Assessment of Catatonia

Catatonia is a psychomotor syndrome and its diverse symptomatology can include motor, mental, behavioral, and vegetative manifestations (Madigand et al., 2016). Its broad clinical spectrum can be classified into the motor and psycho-affective symptoms. Motor manifestations encompass mutism, stupor, staring, grimacing, stereotypy, mannerisms, perseveration, withdrawal, impulsivity, combativeness, cessation of self-initiated movement, and mitgehen and/or gegenhalten reflexes. Psychoaffective symptoms include automatic obedience, verbigerations, echopraxia, repetitive speech, and refusal to eat and drink (Tandon et al., 2013).

One possible categorization of catatonia variants is the one proposed by Fink and Taylor, who argue this syndrome can occur in three forms: retarded, excited, and malignant (Fink and Taylor, 2009). The most recognized is the retarded form, characterized by immobility, posturing, mutism, stupor, and stereotypy. The excited form includes restless movements, talkativeness, agitation, frenzy, and delirium. Lastly, and less commonly found, there is a malignant form, distinguished by autonomic excitement, abnormal blood pressure, tachycardia, and tachypnea (Mashayekhi and Ghayoumi, 2019). The latter resembles many aspects of the neuroleptic malignant syndrome (NMS) and must be strongly taken into account as it might result in death (Philbrick and Rummans, 1994; Ghaziuddin et al., 2017).

Clinical manifestations of catatonia are extremely heterogeneous, making its recognition challenging. There are many scales designed to diagnose catatonia that mainly focus on clinical symptoms. The DSM-V, BFCRS, Brauning, and Northoff scales are summarized and contrasted in Table 1 (Northoff et al., 1999a; Sienaert et al., 2011; American Psychiatric Association, 2013; Aandi Subramaniyam et al., 2020). It is important to differentiate catatonia from other conditions such as nonpsychiatric stupor, encephalopathy, stroke, stiff-person syndrome, Parkinson’s disease, locked-in syndrome, malignant hyperthermia, nonconvulsive status epilepticus, NMS, or autism (Ohry, 1990; Tormoehlen and Rusyniak, 2018; Apetauerova et al., 2021; Vaquerizo-Serrano et al., 2021). Underlying medical conditions might be the cause of catatonia and their treatment should resolve the catatonic syndrome.

**Table 1:** Diagnostic scales comparison: Bush–Francis Catatonia Rating Scale (BFCRS), Rogers Catatonia Scale (RCS), Braunig Catatonia Rating Scale (BCRS), Northoff Catatonia Rating Scale (NCRS).

**Items**	***BFCRS* (Bush et al., 1996**	***RCS* (Sienaert et al., 2011)**	***BCRS* (Bräunig et al., 2000)**	***NCRS* (Northoff et al., 1999a)**
**Excitement**	X	X	X	X
**Immobility/stupor**	X	X	X	X
**Mutism**	X	X	X	X
**Staring**	X			X
**Posturing/catalepsy**	X	X	X	X
**Grimacing**	X		X	X
**Echopraxia/echolalia**	X	X	X	X
**Stereotypy**	X	X	X	X
**Mannerisms**	X	X	X	X
**Verbigeration**	X	X	X	X
**Rigidity**	X		X	X
**Negativism**	X	X	X	X
**Waxy flexibility**	X	X	X	X
Gegenhalten	X		X	X
Mitgehen/Mitmachen	X	X	X	X
Abnormal speech		X		X
Dyskinesia/parakinesia		X	X	X
Iterations		X	X	
Slowness/feebleness of spontaneous movements		X		
Simple abnormal posture		X		
Gait: reduced associated movements		X		
Gait: slow/shuffling		X		
Automatic obedience	X		X	X
**Impulsivity**	X		X	X
Grasp reflex/grasping	X		X	
Akinesia			X	X
Festination/jerky movements			X	X
Rituals			X	
Combativeness/aggression	X			X
Autism/withdrawal	X			X
Ambivalence/Ambitendency	X			X
Perseveration	X			X
Autonomic/vegetative abnormality	X			X
Agitation				X
Flaccidity/muscular hypotonus				X
Affect-related behavior				X
Affective latence				X
Flat affect				X
Anxiety				X
Athetotic Movements				X
Compulsive behavior				X
Compulsive emotions				X
Emotional lability				X
Sudden muscular tone alterations				X
Increased, compulsive-like speech				X
Loss of initiative				X
Magnetism			X	

## Hallmarks of Motor and Psycho-Affective Neural Circuits Involved in Catatonia

In this section, based on the clinical division of catatonia, we describe in detail the physiological functioning of neural circuits related to motor control and psycho-affective modulation.

### Motor control pathways

As previously described, catatonia has a strong motor component, and, therefore, it is mandatory to understand the circuitry implicated in movement control and coordination and its dysfunction in this syndrome. Basal ganglia are collections of neuronal nuclei located in the brain that encompass structures such as the striatum, composed of the caudate nucleus (CN) and putamen, as well as the ventral striatum (VS), internal (iGP) and external globus pallidus (eGP), substantia nigra pars compacta (SNc) and reticulata (SNr), and the subthalamic nucleus (STN). Interconnectivity between these structures and the cerebral cortex, cerebellum, and thalamus is crucial for movement control. The direct and indirect pathways describe how basal ganglia either initiate or completely terminate movement (Graybiel et al., 1994; Lanciego et al., 2012; Bostan et al., 2018).

The direct pathway starts when the cerebral cortex sends projections to the striatum to initiate movement. This excitatory efference activates the GABAergic medium spiny neurons in the caudate and putamen, which project to the iGP and the SNr, resulting in the inhibition of both structures (Calabresi et al., 2014). Tonic inhibitory neurons arising from the iGP would inhibit the ventral lateral (VL) and the ventral anterior (VA) nuclei of the thalamus; however, as iGP activity ceased due to the previous inhibition by the striatum, it is unable to inhibit the VA/VL thalamic complex, leaving the thalamus activated and thus allowing the afference originally initiated in the cerebral cortex to finally reach the frontal cortex, translating these signals into movement (Báez-Mendoza and Schultz, 2013; Cui et al., 2013; Freeze et al., 2013).

On the other hand, the indirect pathway seeks to modulate the disinhibitory actions from the direct pathway. The activation of this pathway is marked by the integration of cortical afferences in the striatum, where spiny neurons coming from the striatum will synapse with the tonic inhibitory active neurons from the eGP, which will later project to the iGP and the STN (Smith et al., 1998). This interaction between the striatum and eGP inactivates the nucleus’ ability to inhibit the STN. Consequently, the subthalamic nucleus neurons project and activate the iGP, which modulates the VL/VA thalamic nuclei, suppressing its activity and thus blocking the activation of the thalamus, leading to termination of movement.

Movement is also inhibited by the hyperdirect pathway, which starts with an excitatory efference arising from the cerebral cortex that projects to the STN. Once activated, the STN projects excitatory afferences to activate the iGP. As has been stated, when the iGP medium spiny neurons project to the VL and VA nuclei of the thalamus, the activation of the medium spiny neurons will inhibit the aforesaid nuclei, leaving them incapable of sending excitatory signals to the cortex, which translates into the inhibition of movement (Nambu et al., 2002).

**Figure 1:**
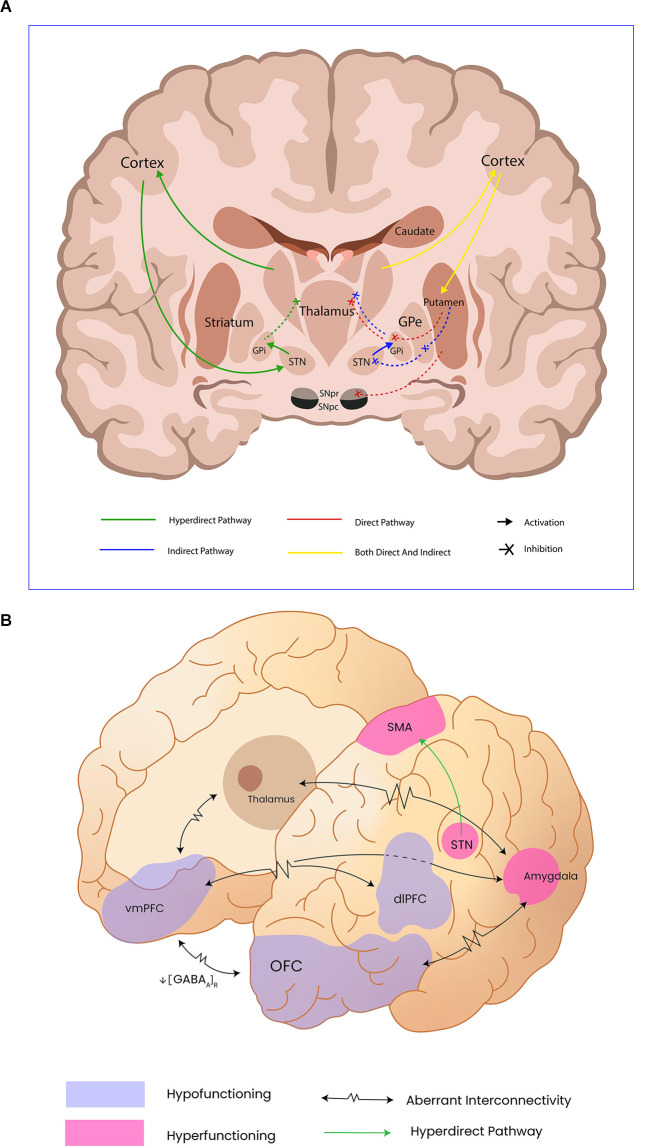
Panel **(A)** shows motor control pathways and the cortical and subcortical structures involved in movement control; Internal Globus pallidus (GPi), External Globus Pallidus (GPe), Subthalamic nucleus (STn). Panel **(B)** illustrates the abnormal functioning of cortical and subcortical structures reported by functional neuroimaging as well as the aberrant connectivity in catatonia; Orbitofrontal cortex (OFC), ventromedial prefrontal cortex (vmPFC), and dorsolateral prefrontral cortex (dlPFC) have been addressed as hypofunctioning, probable due to diminished GABAa receptor density. Subthalamic nucleus (STn), supplementary motor area (SMA), and the amygdala remain hyperfunctioning in catatonic patients, leading to motor inhibition.

Medium spiny neurons from the striatum project directly to the SNc, which also sends diffuse dopaminergic projections to the spiny neurons. The effects dopamine has over spiny neurons are complex and might just be an example of how the same neurotransmitter has a different effect depending on the type of receptor the postsynaptic neurons express. In this sense, the same nigral neurons might provide excitatory afferences to the medium spiny neurons projecting to the iGP, thus promoting movement (direct pathway); but at the same time, these neurons could also provide inhibitory afferences to the medium spiny neurons projecting to the eGP, hence inhibiting movement. The expression of dopamine type 1 receptors (D1R) on the medium spiny neurons in the direct pathway guarantees this excitatory effect, whereas the expression of the dopamine type 2 receptor (D2R) on the same kind of neurons in the indirect pathway has an inhibitory effect (Gerfen and Surmeier, 2011). Figure 1A illustrates these pathways.

### Psycho-affective modulation circuits

Catatonia comprises not only motor but also affective and behavioral abnormalities. Therefore, in this section, we describe the neural substrate underlying emotional processing and the genesis of psychosis.

Regarding the neurobiological correlates of affective processing, it is crucial to understand catatonic affective symptoms by first explaining emotional regulation. Emotions can be defined as physiological responses to a given stimulus. The central processing of emotions is carried out by subcortical structures such as the amygdala, the striatum, the hypothalamus, the hippocampus, and the brain stem; cortical structures comprise the somatosensory cortex, cingulate cortex, and the motor cortex (Damasio and Carvalho, 2013). The amygdala is a key structure implicated in positive and negative emotional processing. Sensory inputs from the thalamus arrive at the lateral nucleus of the amygdala, connecting therefrom to the central nucleus, which in turn projects to the central gray region, and to the lateral and the paraventricular hypothalamus. Efferences to the autonomic and limbic systems result in an endocrine and motor output, usually evident as facial expressions, or more complex motor behaviors like freezing or running (Yang and Wang, 2017).

The genesis of psychosis has been tightly related to dopamine dysfunction. The classical dopaminergic theory stated there was an overactivity of the dopamine pathways due to enhanced neurotransmission or an increased sensibility to dopamine (Seeman and Seeman, 2014). Nonetheless, the role of dopamine in psychosis has evolved from a general hyperdopaminergic state to a complex dysregulation, where there is greater activity of dopamine in the mesolimbic pathway and a lower dopaminergic activity in the mesocortical pathway (Kumakura et al., 2007; McCutcheon et al., 2020).

The increased sensibility to dopamine in the mesolimbic tract is thought to rely on different affinity states for agonism of D2R (Seeman et al., 1975; Seeman, 2013a). The different affinities are inter-convertible and depend on the coupling with G-proteins, where a coupled state is a high-affinity state (D2High) while the uncoupled state is a low-affinity state (D2low; Graff-Guerrero et al., 2009). Seeman et al. (2006) demonstrated that in schizophrenia there is an increase in D2High which is associated with a greater sensitivity to dopamine.

Our understanding of dopamine has evolved from its classical function as a reward neurotransmitter to having a more complex spectrum of functions including valuation, decision making, motivation behavior, awareness, and prediction of reward (Schultz et al., 1997; Fusar-Poli et al., 2010). Midbrain dopaminergic neurons and their interactions with other CNS structures have been postulated to mediate motivation, behavior, and valuation, and an aberrant circuitry function might be the substrate to aberrant behaviors (Montague et al., 2004). Furthermore, under normal conditions, dopamine is essential to make an accurate internal representation of an external stimulus. Thus, when altered, an incongruent representation and interpretation of the external stimulus results in psychosis (Howes and Kapur, 2009; Fusar-Poli et al., 2010; Howes et al., 2013). The *Aberrant Salience Theory* states complex diseases such as SCZ emerge from an incorrect adaptation process as a consequence of functional abnormalities in dopamine-related pathways (Roiser et al., 2013; Howes and Nour, 2016).

In brief, emotional regulation is performed by cortical and subcortical structures, and autonomic and motor responses are consequences of these processes. The genesis of psychosis is related to dopamine dysregulation, affecting the articulation of external vs. internal stimuli. The aforementioned structures and neurotransmitters juxtapose with those affected in catatonia. Furthermore, alterations in these circuits sometimes present with similar symptoms to those seen in catatonia.

## Pathophysiology of Catatonia

### The role of neurotransmitters and circuitry dysfunction

In this section, we review and aim to elucidate catatonia’s physiopathology by articulating functional imaging findings, preclinical data, current theories regarding neurotransmitter dysfunction, and successful pharmacological treatments reported in the literature. Understanding the aforementioned voluntary control movement, affective and behavioral neural substrates is crucial to correlate findings.

Recent systematic evidence (Haroche et al., 2020) gathers non-homogenous findings regarding structural imaging in patients diagnosed with catatonia. The most common cortical areas associated with catatonia are M1, the SMA, the parietal cortex, and the ventromedial prefrontal cortex (vmPFC). The frontal and parietal cortices are consistently found diminished, especially the right medial orbitofrontal cortex (OFC) and the superior left parietal gyrus (SPG). Disparate findings encompass fronto-parietal cortical sulcal enlargement, hyper gyrification of the anterior cingulate gyrus, medial OFC, right inferior temporal gyrus, and right insula, as well as hypo gyrification in the left superior temporal gyrus. Other important but not extensively studied structural findings are cerebellar vermis and brainstem atrophy and increased gray matter density in the cerebellum cortex (Wilcox, 1991; Northoff et al., 1999c).

The SMA is strongly related to the initiation, planning, learning, and programming of motor behavior, while the main synaptic outputs related to motor control encompass STN and parietal cortex stimulation. It is also associated with the integration of sequential elements into higher-order representations in complex processes like music, language, and working memory, implying that any required sequential process is structured by the SMA. In non-catatonic patients, SMA lesions manifest as slurred speech, perseverations, and repetitions. The radical cessation of self-initiated movement and sequence of movements are also observed (Ziegler et al., 1997).

Walther et al. (2017) found schizophrenic patients with catatonia had an increased cerebral blood flow to the SMA that directly correlates to catatonia severity. Moreover, they highlighted a diminished gray matter density in the insular and frontal cortices in these patients. A study performed by J. Scheuerecker found a normal medial motor loop (SMA, thalamus, and basal ganglia) activation in healthy control patients while performing self-initiated movements, but no activation in patients with catatonic schizophrenia (Scheuerecker et al., 2009). Additionally, regarding stimulation of the hyperdirect pathway, the VIIb cerebellar region projects to the STN, which in turn blocks movement by inhibiting M1 and the SMA (Walther et al., 2017). Taken together, the SMA integrates motor and cognitive information. Experimental and clinical SMA alterations in non-catatonic patients resemble motor and psychoactive symptoms observed in catatonia. The remarkable hyperperfusion of the SMA found on functional imaging in catatonic patients might correspond to compensation for insufficient or inhibitory basal ganglia output, and increased neural activity to override massive motor inhibition, probably coming from the cerebellum and the STN.

Another important finding using statistical functional imaging analysis is that catatonic patients present significant alterations in connections between the OFC and the vmPFC, and between the ventromedial/dorsolateral PFC and the premotor/motor cortex. Moreover, functional imaging studies showed a decrease in cerebral blood flow in the right PFC and parietal cortex as well as a decrease in GABA-A receptor density and binding in the left sensorimotor cortex and right and left lower PFC (Northoff et al., 1999b). Northoff proposes a “top-down” modulation model in which he explains akinesia as a downregulation of the direct motor loop, emphasizing the fact that the source of this dysregulation does not come from the motor loop *per se* but rather from the GABAergic and cortico-cortical dysfunctions mediated by horizontal modulation in OFC and PFC. Vertical modulation understood as bidirectional interconnections between cortical and subcortical structures has also been suggested to explain motor symptom expression (Northoff, 2002; Hirjak et al., 2015).

Furthermore, vmPFC-thalamic connections are also crucial in cognitive behavioral control. Divergent projections from the vmPFC and dmPFC arrive at the mediodorsal thalamus and the ventromedial striatum, and apparently, these circuits maintain cognitive control tasks and guide a proper behavioral output by establishing a constant representation of a given task (de Kloet et al., 2021). The regulation and tracing of these interconnected projections are quite complex: there are at least four different circuits linking the vmPFC and dmPFC to the thalamus and striatum. Concerning catatonia, inhibitory corticostriatal projections arising from the mPFC to the striatum have been described; therefore, as seen in functional images, vmPFC and dmPFC hypofunction could activate striatum medium spiny neurons, resulting in behavior arrest (Lee et al., 2014).

Common associative areas are found when contrasting motor control, emotional regulation, and the genesis of psychosis. Subcortical structures like the basal ganglia circuits and the amygdala are involved in behavioral and emotional regulation, as well as cortical associative structures such as the primary motor cortex M1, SMA, PFC, OFC, thalamus, and the cerebellar-thalamo-cortical circuit (Walther et al., 2017). Functional MRI studies allow us to think of catatonic behavior as the result of a negative emotional processing, where a hyperactivation of the amygdala and in contrast a decreased activation of the OFC, supramarginal gyrus (SMG), mPFC, and cingulate anterior cortex (CAC) result in motor activity suppression, or tonic immobility as a response to fear (Northoff, 2002; Ellul and Choucha, 2015; Hirjak et al., 2019a).

Building on these ideas, Northoff and Hirkjak (Northoff et al., 2004; Hirjak et al., 2019b) both showed that a decreased activation and area of the OFC were related to behavioral and affective symptoms in catatonic patients, whereas motor abnormalities were linked to mPFC dysfunction. Catatonic patients have subjectively reported feeling overwhelmed with emotions, predominantly negative ones and specifically anxiety. Accordingly, depression can present as a prodrome of catatonia, while anxiety and psychotic symptoms can be present as well.

Additionally, catatonia might be considered as a fear syndrome, with cataplexy resembling a prey’s freezing response while being hunted by a predator (Moskowitz, 2004). This behavior is related to an animal defense strategy named tonic immobility, which consists of sudden freezing when the animal is exposed to a dangerous stimulus (Lander et al., 2018). Environmental stimuli are conveyed in different brain areas that later project to the amygdala. After the information is sent to the amygdala, different cortical areas are activated. For example, connections between the OFC and the amygdala are assumed to be involved in emotional processing and control, especially when it comes to negative emotions. In consequence, decreased activation of the OFC impedes inhibition of the amygdala, thus leading to hyperactivation of the latter (Rempel-Clower, 2007; Ellul and Choucha, 2015). The vmPFC is a key area for fear generalization and perceiving emotions as well. Interesting evidence frames vmPFC interconnectivity between the amygdala and thalamus as a key regulator of fear and anxiety, so a reduced thickness or misfunction might lead to generalized anxiety (Arruda-Carvalho and Clem, 2014; Cha et al., 2014; Motzkin et al., 2015). Altogether, it is clear the OFC and vmPFC are key regulators of emotional and behavioral processing, and alterations seen in other pathologies help us shed light upon imaging findings in catatonia.

Symptoms such as echolalia and echopraxia are frequently present in catatonic patients. Previous studies have shown imitation behaviors might arise in response to lesions in the OFC. These echo-phenomena arise from the disinhibition of mirror neurons as a result of dysfunction in the GABAergic system within the OFC (Mehta et al., 2013; Ellul and Choucha, 2015).

The cellular and molecular mechanisms underlying the above-mentioned phenomena are scarce. Explanatory hypotheses are based on pathway dysfunction rather than a focal lesion in the central nervous system. The main neurotransmitters considered to play a critical role in catatonic pathophysiology are glutamate, GABA, and dopamine (Northoff et al., 1999b; Rasmussen et al., 2016). Akinetic catatonia, characterized by stupor, might be explained based on hypo functioning of GABAergic receptors, either by intrinsic dysfunction or by the overriding of said pathway generated by excess glutamate or NMDA receptor activity (Northoff et al., 1997; Carroll et al., 2007; Wijemanne and Jankovic, 2015). This hypothesis is supported by evidence showing catatonic responders to lorazepam developed hypokinetic movements, rather than hyperkinetic ones (Northoff et al., 1995). Serotonin plays a crucial role in indirect motoric control as the raphe nucleus directly modulates the activity of VTA and SN, and therefore less dopamine is released into the subcortical-cortical motor-related structures. In essence, the greater the activity of the raphe nucleus, the lower the activity of dopamine. This regulation is worth considering in psychiatric pathologies where catatonia might be present, as seen in bipolar disorder and major depression, diseases where serotonin is augmented or diminished respectively (Conio et al., 2020; Martino et al., 2020; Northoff et al., 2021).

Interestingly, 72% of all autoimmune cases of catatonia are attributable to NMDAR encephalitis, a disease in which autoantibodies against the N1 subunit of NMDAR are internalized into the cells, leading to a decrease in the total number of receptors, increasing extracellular glutamate concentrations and interrupting synaptic connectivity (Rogers et al., 2019). It might seem counterintuitive that NMDA inhibition relieves catatonic symptoms when their internalization might produce them. Nonetheless, even when we can’t pinpoint an exact cause of why anti-NMDA encephalitis produces catatonia, recent evidence helps us decipher this puzzle. Hare et al. (2020) administered ketamine and ketamine metabolites into the vmPFC of mice to assess their antidepressant actions. Amazingly, they found that the administration of these NMDA inhibitors increases vmPFC function in a dose-dependent manner. It is not clear why, but NMDA inhibitors probably modulate gene expression of calcium/calmodulin-dependent protein kinase II alpha (CaMK2A), a subunit intrinsically related to NMDA synaptic plasticity and long-potentiation. This allows us to infer that the administration of NMDA inhibitors, by modulating pyramidal CaMK2A-expressing neurons, can modify and reshape NMDA-dependent synapses in the vmPFC making them more active, nonetheless, more research needs to be done to verify this hypothesis. Catatonic patients who are not fully responsive to Benzodiazepines (BDZ) have shown positive response to treatment in combination with NMDA inhibitors such as amantadine and memantine (Babington and Spiegel, 2007; de Lucena et al., 2012; Ellul and Choucha, 2015). Table 2 summarizes some cases reported in the literature using amantadine in combination with other medications resulting in positive clinical outcomes.

**Table 2:** Successful cases reported using amantadine, therapy regimen, and outcome.

**Patient’s diagnosis**	**Reference**	**Number of patients**	**Therapy regimen**	**Outcome**
Bipolar disorder and catatonia	Ene-Stroescu et al. (2014a)	1	Amantadine (Dose notdisclosed) + Lorazepam + Carbamazepine	Full response after 72 h
Catatonia	Goetz et al. (2013)	1	ECT + Amantadine 250 mg/day	Discharged after 6 weeks. Full recovery
Catatoniain schizophrenia and schizo affective disorder	de Lucena et al. (2012)	5	Case 1: Clozapine + 400 mg of amantadine Case 2: Amantadine protocol Case 3: Amantadine protocol Case 4: Amantadine protocol Case 5: Amantadine protocol *Protocol: up to 600 mg of amantadine daily for 4 weeks	Case 1: Recovery on the 14th day with clozapine Case 2: Recovery by the 10th day Case 3: Recovery at the end of the protocol Case 4: Full recovery on the 8th day Case 5: Acceptable behavior by the 45th day
Catatonia and schizophrenia	Babington and Spiegel (2007)	1	Lorazepam+chlorpromazine+Amantadine 200 mgday	Fast recovery after 48 h of amantadine
Westphal variant Huntington disease and refractory catatonia	Merida-Puga et al. (2011)	1	Antipsychotics + ECT+L-dopa + Amantadine 300 mg/day	Not available
Catatonia and depressive disorder	Hervey et al. (2013)	1	Lorazepam + amantadine 200 mg/day	Recovery once amantadine was initiated
Catatonia, schizophrenia, and progressive diffuse cerebral atrophy	Ene-Stroescu et al. (2014b)	1	Clozapine+lorazepam+divalproex+amantadine (dose not specified)	Maximal response after 8 weeks of treatment
Catatonia in high-functioning ASD	Ellul et al. (2015)	1	Initial regimen of Zolpidem+lorazepamthen a 1-week washout followed by amantadine 200 mg/day	Maximal recovery after 2 weeks with amantadine
Akinetic catatonia	Northoff et al. (1997)	3	Case 1: Three IV infusions of amantadine 500 mg/dose Case 2: Two IV infusions of amantadine 500 mg/dose Case 3: Two IV infusions of amantadine 500 mg/dose	Case 1: Recovery after the third infusion of amantadine Case 2: Recovery after the second infusion Case 3: Recovery after the third infusion	#x02014;needed lorazepam to treat aggressive and anxious behavior

As for dopamine, the dopaminergic D2 blockade provided by first-generation antipsychotics has been commonly associated with Neuroleptic-Induced Catatonia. Neuroleptics induce a cataleptic state in rodents; this state is also called “animal catatonia”, and it is used to measure propensity to extrapyramidal effects and in relation to D2 blockage (Lee, 2010; Ghaziuddin et al., 2017). As for dopaminergic signaling, the exact mechanism by which a dysregulation might cause catatonic signs is not clearly elucidated (Daniels, 2009). Treatment response, either improving symptoms or provoking them, shines a light as to which pathways and neurotransmitters are involved in catatonia.

Although current treatment regimens for catatonia will be discussed in Section “Revisiting current therapeutics”, it is essential to examine the scenario of withdrawal catatonia. Few but consistently reported cases show that abrupt discontinuation of BDZs and antipsychotics, more often clozapine, is followed by catatonia (Brailey and Bastiampillai, 2020; Belteczki et al., 2021). These cases strengthen the evidence regarding the involvement of GABA receptors and dopamine circuit dysfunction in catatonia‘s physiopathology, but the question of how this happens remains. Some authors argue that the downregulation of GABA receptors due to long-term use of these medications might be the mechanism of withdrawal catatonia (Lander et al., 2018), which would concordantly relate to the aforementioned evidence. Nonetheless, it is worth considering that clozapine has unique mechanisms of action, is a D2 fast-off antipsychotic (Seeman, 2013b), an antagonist of 5HT2 and α2-adrenoceptor, and an indirect agonist of NMDAr, as it increases glycine synaptic availability by blocking the SNAT2 (Wenthur and Lindsley, 2013). In this regard, we would add to the underlying cause of catatonia onset shortly after abrupt cessation of clozapine the downregulation of D2 receptors, increased 5HT2A activity, and decreased NMDAr function.

Functional neuroimaging findings have been presented throughout this section, the main cerebral regions involved being the SMA, vmPFC, and OFC, and the current hypotheses involving neurotransmitter dysfunction have been discussed as well. Different authors have correlated these findings with the clinical spectrum of catatonia; however, the overlapping of motor and behavioral findings, added to the clinical heterogeneity, makes it difficult to come to a conclusive finding when it comes to the study of catatonia. As for the OFC, hyperactivation, and hypoactivation have been found in different studies: hyperactivation of the OFC correlated directly with negative emotional and affective processing in catatonic patients (Ellul and Choucha, 2015), while decreased activation of the OFC has been documented as well and has been associated with tonic immobility, one of the catatonia’s pathophysiological theories (Northoff et al., 2004). Moreover, another key finding in catatonia is hypofunction of the vmPFC, which, as explained, has diverse projections to the amygdala, basal ganglia, and other subcortical structures related to fear extinction and behavioral control, and therefore its dysfunctions might be directly related to catatonic symptoms. On the other hand, SMA findings have been more homogeneous, indicating a directly proportional relationship between increased rCBF and clinical severity; however, discrepancies in blood flow have been seen depending on the clinical subtype of catatonia. For example, retarded-state catatonic patients were documented to have an overly activated SMA when compared to the excited subtype, linking increased neural activity to massive motor inhibition (Walther et al., 2017). Importantly, cortico-basal ganglia-thalamocortical loops related to motor, behavioral and emotional processing are the common substrate of a wide variety of symptoms present in catatonia. We consider that the disparity between objective findings in neuroimages relies on the diverse scenarios in which catatonia presents, and strengthens the theory that catatonia is the result of diffuse dysfunction rather than a focal lesion in the CNS. Figure 1B illustrates and contrasts with normal motor circuits and alterations evidenced in catatonic patients.

### Neuroinflammation in catatonia

In this section, we present the underlying cellular processes occurring during catatonia, especially focusing on the role of glia. Catatonia onset has been frequently associated with proinflammatory environments, whether as a trigger or comorbidity (Goforth, 2007; Quinn and Abbott, 2014). Inflammatory mechanisms leading to catatonia can only be hypothesized as current evidence is still insufficient to address causes. Nonetheless, we here present research evidence extracted from interesting animal models to help understand the roles of microglia, astrocytes, and oligodendrocytes in catatonia, and therefore how inflammation and oxidative stress could lead to this disease.

Microglia are CNS-resident immune cells actively involved in defense, pruning, and scar formation, and which are constantly interacting with other resident cells such as oligodendrocytes, astrocytes, and neurons (Liu et al., 2020). Early postnatal migration of microglia supports myelination and full synaptic functionality by inducing oligodendrocyte progenitor cell maturation through neurotrophic factor secretion when exposed to interleukins (IL) 4, 10, 13, and 33 (Butovsky et al., 2006; Banisadr et al., 2011; Miron et al., 2013). Microglia are also a key factor for remyelination: studies showed that an M2 (anti-inflammatory) rich environment prompts oligodendrocyte remyelination (Hagemeyer et al., 2017), while an M1 microglia (proinflammatory) rich environment enhances interleukin production, increasing antigen presentation and oxidative stress, and blocking M2 myelination properties (Miron et al., 2013). Moreover, the production of Tumour Necrosis Factor alpha (TNF alpha), nitric oxide (NO), and complement due to microglia activation are shown to induce oligodendrocyte death and phagocytosis (Greenhalgh et al., 2020).

2’,3’-cyclic nucleotide 3’-phosphodiesterase (CNP) is an oligodendrocyte membrane enzyme protein. It has two isoforms, the first of which is related to RNA, tubulin, calmodulin and actin binding, ATP/GTP hydrolysis, and catalytic activity. These multiple functions are postulated to bridge the gap between the cytoskeleton and membrane, and are probably related to the transport of cargo along the axon, as well as the activation of second messenger (Raasakka and Kursula, 2020). The second isoform is found in mitochondria, and when activated it opens the transition pore, leading to cellular apoptosis (Krestinina et al., 2015). CNP also oversees the catalysis of 2’,3’-cyclic nucleotides and it is thought to prompt the expression of other structural myelin proteins in underdeveloped oligodendrocytes (Scherer et al., 1994; Myllykoski et al., 2012). Rodents null ^(−/−)^ for Cnp develop catatonia with aging, as soon as 8 weeks old (Janova et al., 2018). Cnp-heterozygous ^(+/–)^ rodents develop behavioral abnormalities that from a translational perspective resemble behaviors seen in schizophrenia and depression (Hagemeyer et al., 2012). Although evidence pinpointed Cnp as a feasible genetic mutation related to schizophrenia and catatonia, association studies did not support this correlation (Tang et al., 2007; Che et al., 2009). It is interesting that null and heterozygotic mice show behavior resembling the motor and affective symptoms characteristic in schizophrenia and catatonia. Furthermore, when Cnp−/− mice are exposed to PLX5622, a colony-stimulating factor inhibitor, and therefore a microglia suppressor, catatonia is ameliorated or prevented (Janova et al., 2018). These findings lead us to think microglia and oligodendrocytes play a role in catatonia.

When it comes to catatonia, evidence shows systemic pro-inflammatory environments trigger microglia, as well as metabolically stressful situations (Wolf et al., 2017). Even with subtle activation, a proinflammatory reaction of microglia can swell or damage the myelin sheet, generating a connectivity dysfunction (Poggi et al., 2016). We can infer the reaction of microglia in catatonia, as preclinical evidence has shown increased inflammation and myelin sheet swelling in the anterior corpus callosum and PFC, anatomical structures deeply correlated to affective and motor circuits, as described above (Hagemeyer et al., 2012; Janova et al., 2018). Although available preclinical evidence sheds light on catatonia development, the processes that determine the onset of this complex syndrome are not clear.

Astrocytes play a critical role in myelination, specifically through connexins that together form gap junctions: these proteins allow the diffusion of ions and molecules smaller than 1.5 kDa (Nagy and Rash, 2003; Orthmann-Murphy et al., 2008; Liang et al., 2020). In the CNS, astrocytes express connexins 26, 30, 46, and 47, and connect to oligodendrocytes through connexins 47/43, 47/30, 32/26, and 32/30. It is worth noticing that astrocytic connexin 47 is exclusive to the corpus callosum, striatum, cerebellum, and spinal cord. Astrocytes modulate myelination by providing oligodendrocytes with lipids and adjusting the structure and conduction velocity of myelin by modulating potassium homeostasis (Camargo et al., 2017; Sock and Wegner, 2019; Xia et al., 2020). Microglia directly modulate astrocyte function through the expression of IL-1B, IL-6, and TNF, turning them into a cytotoxic form characterized by loss of astrocytic gap junctions, increased glucose uptake, intracellular trafficking restriction, increased expression of complement, and poor stabilization and shaping of synapses (Prinz and Priller, 2014; Greenhalgh et al., 2020). Once astrocytes are turned into pro-inflammatory A1 type, astrogliosis is initiated and therefore neuroinflammation is perpetuated, thereby impeding normal synaptic connectivity. We consider astrocytes to play a role in catatonia, as Hagemeyer et al. (2012) showed high astrogliosis in the corpus callosum in CNP-heterozygous mice.

### Oxidative stress in catatonia

Oxidative stress (OS) is a well-known consequence derived from an imbalance between the production of antioxidants and reactive species (RS), the latter group encompassing reactive oxygen (ROS) and nitrogen species (RNS). ROS include hydrogen peroxide (H_2_O_2_), peroxyl radicals (ROO·), the superoxide anion radical (O_2_·(-)), singlet molecular oxygen (O_2_^-^), and the hydroxyl radical (OH·). RNS encompass peroxynitrite (ONOO^−^) and NO (Sies, 2015). Naturally produced RS are buffered by potent antioxidants like superoxide dismutase (SOD) and glutathione (GSH). In pathological conditions, the increase in RS surpasses the production of antioxidants, and therefore RS are freed into the environment, reacting with membranes, damaging nuclear and mitochondrial DNA, and finally inducing cells to apoptosis (Qi and Dong, 2021). It is worth noting that the brain is particularly susceptible to OS, since for its physiological functioning it consumes as much as 20% of the body’s oxygen supply, and is, in a great proportion, formed by lipids; therefore, in homeostatic environments, abundant RS are produced but they are naturally dampened by antioxidants produced by the mitochondria.

When it comes to catatonia, it is worth considering OS as a key factor, as the perphenazine-induced catatonia mice model has shown increased OS and depletion of antioxidant concentration when compared to controls. Interestingly, pretreatment with the cyclooxygenase-2 selective inhibitors rofecoxib and celecoxib decreased catatonic behavior, lipid peroxides, and brain nitrite (Gupta et al., 2009, 2011). Furthermore, it is widely known now that OS can directly activate multiple pathways related to inflammation. OS triggers the receptor for advanced glycation end-products (RAGE) and nuclear factor kappa-light-chain-enhancer of activated B cells (Nf-KB), leading to increased production of pro-inflammatory cytokines which will consequently create positive feedback between microglia and astrocyte activation (Kouidrat et al., 2013; Qin et al., 2013; Lingappan, 2018).

### The glial syncytium in catatonia

The neurons, astrocytes, and oligodendrocytes connect through gap junctions, also known as the glial syncytium, thought to improve synaptic connection, calcium signaling, and regulate metabolism as well as cellular trafficking (Orthmann-Murphy et al., 2008; Xia et al., 2020). Bearing in mind the specific roles of each cell in the prior sections, a dysfunction of any of them can cause a broader alteration of this syncytium.

We consider neuroinflammation as a key process underlying catatonia as acute ill patients diagnosed with autoimmune diseases or infections sporadically present with catatonic symptoms (Cawkwell et al., 2021; Zain et al., 2021; Sakhardande et al., 2022). Microglia and astrocytes are sensitive to inflammation, its morphological change to M1-A1 respectively is known to cause connexins to decouple, neurons and oligodendrocytes injury by activation of complement and oxidative stress, and thus generating weak synaptic transmission, myelin destabilization, and excitatory-inhibitory imbalance (Orthmann-Murphy et al., 2008; Verkhratsky et al., 2009; Verkhratsky, 2010). On the other hand, some cases reported using different medications, whether to treat psychosis, and immunomodulate, have been linked to the onset of catatonia (Bhangle et al., 2013; Lander et al., 2018; Durns et al., 2020). These clinical strengths the hypothesis that catatonia is predominantly an immune disease, and disturbing astrocytes and microglia directly interrupt movement control neural networks. Moreover, inflammation and OS directly damage the blood-brain barrier (BBB), creating a non-hermetic sealing of the brain and therefore allowing the leakage of plasma, proteins, and inflammatory cells to the brain parenchyma, affecting the homeostatic environment of the glial syncytium (Varatharaj and Galea, 2017).

**Figure 2:**
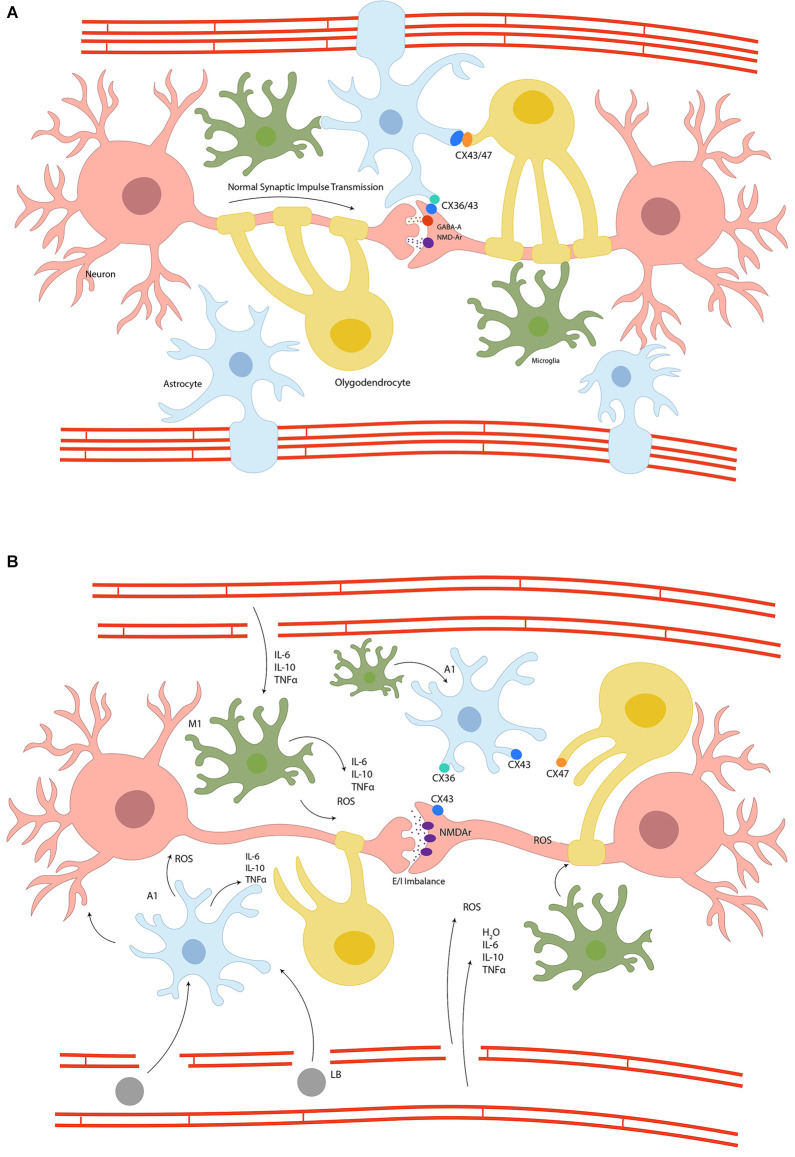
Panel **(A)** represents the glial syncytium, where astrocytes, oligodendrocytes, and neurons are coupled through connexins to stabilize the synapsis and the blood-brain barrier, preserving homeostasis and therefore a normal synaptic impulse transmission. Panel **(B)** schematizes the disruption of glial syncytium due to inflammation and metabolic dyshomeostasis. The switch of glial cells to proinflammatory phenotype results in connexins uncoupling, impedes synaptic stability and an excitatory/inhibitory imbalance, the leaking of blood-brain barrier and therefore migration of systemic inflammatory cells, increase oxidative stress, and the activation of apoptotic cascades ending in cellular death; Connexins (CX), N-methyl-D-Aspartate Receptor (NMDAr), Gamma-Aminobutyric acid-A (GABA-A), lymphocyte B (LB), Interleukin (IL), Tumour Necrosis Factor-alpha (TNFα) reactive oxygen species (ROS), proinflammatory astrocyte (A1), proinflammatory microglia (M1), water (H_2_O).

Taken together, inflammation and OS are hallmarks of catatonia’s physiopathology, as they form a positive feedback loop, creating a non-homeostatic environment in which microglia, astrocytes, oligodendrocytes, and neurons are disturbed, generating weak synaptic transmission. Figure 2 illustrates glial syncytium and its dysregulation in catatonia.

## Revisiting Current Therapeutics

Once catatonia is recognized, pharmacologic interventions and ECT can be used as they have shown the best clinical results so far (Denysenko et al., 2018). Currently, the first line of treatment are benzodiazepines (BDZ), due to their effect on GABA transmission by GABA_A_ receptor agonism (Goodchild, 1993), especially lorazepam, which is the most widely used with a variable dosage ranging from 2 to 16 mg per day. For its part, ECT is effective in all forms of catatonia, even when BDZs have failed. Early intervention with this technique is recommended to avoid deterioration of the patient’s medical condition; indeed acute severe and psychotic mood disorders have shown good therapeutic response to this therapy (Nolen and Zwaan, 1990; Luchini et al., 2015). Other medications such as dantrolene (Pennati et al., 1991), amantadine (Northoff et al., 1997; Hervey et al., 2012), and anticonvulsants (Rankel and Rankel, 1988) are reported as effective therapies in refractory catatonia. This section summarizes the current therapies used in catatonia from a mechanistic perspective.

### Benzodiazepines (BDZ)

BDZs are GABA_A_ allosteric modulators that enhance chloride conductance by binding to the BZ site of the receptor. Increasing the conductance of chloride ions is translated into an increasing frequency of channel opening. Congruently, BDZ efficacy is directly related to GABAergic enhancement (Walther et al., 2019). Some authors attribute the success of BDZ to an induced decreased activation of mPFC and OFC (Edinoff et al., 2021). We support this hypothesis and suggest that a GABAergic potentiation would decrease preSMA-SMA hyperactivation and, moreover, that BDZ could generate diffuse brain circuitry hyperpolarization.

A literature review by Rasmussen et al. (2016) recommends low-dose BDZ therapy (1–2 mg lorazepam SL or IV) followed by a repeated dose every 3 h until therapeutic effects are reached. Doses may be titrated depending on patient age and duration of symptoms, with chronic catatonia requiring higher doses for longer periods. In acute catatonia, 85% of patients had a positive response to treatment while 58.8% of patients recovered within three hours of a single dose of lorazepam. Treatment of the underlying condition must be ensured before discontinuing BDZ to prevent relapse (Rasmussen et al., 2016). Around 27% of patients experience only partial recovery, with poor response predictors being chronicity, increased age, and psychosis (Kritzinger and Jordaan, 2001; Walther et al., 2019).

Flunitrazepam, diazepam, and clonazepam are also viable options. Administration varies depending on the patient’s health condition and collaboration: oral, intramuscular, and intravenous routes are available (Goodchild, 1993; Fink and Taylor, 2009; Pelzer et al., 2018). Recommended doses, titrations, and length of treatment vary among different authors. Reports with low-doses of lorazepam have shown fast responses and symptomatology improvement (Kritzinger and Jordaan, 2001; Sienaert et al., 2014; Rasmussen et al., 2016); however, longer treatments and higher doses have also been reported. A recent systematic review published by Zaman et al. (2019) compared BDZ use in catatonia vs. other drugs, placebo, and ECT, concluding there is not enough high-quality evidence to recommend specific treatment with BDZ, nonetheless BDZ, and regularly lorazepam, remain as the first-line treatment in catatonia, and therefore further research is needed in order to have a standardized regimen for this condition (Korkeila, 2016).

### Antipsychotics

Second-generation antipsychotics are described as an alternative therapy for non-malignant catatonia, especially when a psychotic disorder is suspected as the underlying cause of catatonia. Second- generation antipsychotics block 5HT2A and D2 receptors, which may in turn increase relative dopamine activity in the mesocortical pathway. It is proposed that they may work in catatonia through the treatment of the underlying condition (Sienaert et al., 2019). Even though second-generation antipsychotics might show beneficial effects in catatonia with underlying psychiatric illness, they should be used with caution due to possibility of NMS and other motor side effects.

First-generation antipsychotics are generally not recommended due to the risk of worsening the condition. Several authors suggested that first-generation antipsychotics may aggravate non-malignant and malignant catatonia (Philbrick and Rummans, 1994; Ghaziuddin et al., 2017; Sienaert et al., 2019). Malignant catatonia is diagnosed when there are clear signs of autonomic alteration and hyperthermia, and it must be considered as it is highly lethal (Philbrick and Rummans, 1994). Not to be confused with NMS, which onset is directly related to antipsychotic use.

Beach et al. (2017) performed a systematic review of alternative treatments in catatonia in which aripiprazole, clozapine, olanzapine, risperidone, and ziprasidone were used individually in a total of 33 cases. Most patients recovered in a few days, though recovery was linked to pre-existing schizophrenia. In this study, two patients under clozapine developed catatonia after its withdrawal, which makes its use in this disease controversial. Paparrigopoulos et al. (2009) also described a clozapine-induced neuroleptic malignant syndrome in a patient with catatonia.

A case series described three cases in which intramuscular (IM) aripiprazole was successful in the treatment of catatonia. As an example, the first patient was a 61-year-old male with schizophrenia in his 72nd admission to the mental unit. Acute symptoms were treated with first-generation antipsychotics and BDZ, and his usual PO therapy with clozapine, aripiprazole, and valproic acid was continued. Three days later, catatonic symptoms were evident with a BFCRS score of 52. Iatrogenic catatonia was presumed. Symptoms resolved on the third day of IM aripiprazole at a dose of 10 mg × 3 days (Voros et al., 2009).

Even though success with second-generation antipsychotics is closely linked to pre-existing schizophrenia, it remains elusive whether antipsychotics work by directly resolving the catatonic syndrome or by treating the underlying psychotic disorder associated with catatonia. Stronger evidence is needed to clarify the safety and to standardize a regimen for antipsychotic prescription in patients diagnosed with schizophrenia and catatonia.

### Electroconvulsive therapy (ECT)

ECT is employed in major psychiatric and medical disorders that are refractory to first-line treatments such as mood disorders, Parkinson’s disease, post-stroke depression, schizophrenia, and catatonia. Even though it has been used for decades, the exact mechanism of action through which it is therapeutic is still unknown (Kaliora et al., 2018).

Therapy consists of inducing a controlled seizure under proper sedation. The classical approach includes both temporal lobes, although variations of electrode positions has been reported. In the first session, the initial seizure threshold (IST) is calculated either by administrating a low dose and titrating until a seizure threshold is obtained, or by giving a standard dose and adjusting it depending on the patient’s tolerance considering age, sex, body mass, and co-treatment factors such as the chosen anesthesia (Jeong et al., 2019). Side effects include headaches, myalgias, and memory deficits, which typically resolve within weeks, but may last beyond 6 months in some patients (Lloyd et al., 2020).

ECT is currently the definitive treatment for catatonic syndromes. This complex motor syndrome, as discussed previously, is a potentially life-threatening condition and since ECT’s introduction, the mortality associated with catatonia has decreased. Even though ECT has shown encouraging results in case series studies, there is a lack of high-quality randomized controlled trials to standardize ECT implementation in catatonia, and further research must therefore be carried out to potentiate the benefits of this therapy (Weiner and Reti, 2017). Nonetheless, its broad application with tremendous results, especially those associated with a GABAergic deficit in the orbitofrontal cortex and therefore the top-down catatonia type described by Northoff, has encouraged the use of ECT in catatonia (Northoff, 2002; Luchini et al., 2015).

The mechanisms underlying the success of ECT in catatonia are not elucidated. A neuroendocrine hypothesis has been proposed, but ECT might as well be treating the underlying etiology of catatonia, e.g., bipolar disorder and major depression (Dierckx et al., 2012; Perugi et al., 2017). ECT is thought to act principally in the diencephalon, especially in the hypothalamus where it “restores” endocrine function with the release of various hormones such as adrenocorticotropic and thyroid-stimulating hormones into the systemic circulation and cerebrospinal fluid (Haskett, 2014). Several hypothalamic functions such as sleep, appetite, menstrual cycle, circadian rhythm, and libido are restored after ECT (Fink and Ottosson, 1980; Bodnar et al., 2016; Weiner and Reti, 2017).

Furthermore, ECT has proven to be a safe therapy with positive responses ranging from 80 to 100%, nonetheless, unsuccessful results do not rule out catatonia (Hermida et al., 2018). The mortality rate is 1 in 10,000 patients and it can be increased by medical comorbidities including cardiovascular dysfunction, cerebral disease, and respiratory disease. Though elderly patients may have a higher comorbidity rate, age alone is not considered to increase mortality (Meyer et al., 2018).

### Amantadine and NMDAr antagonists

Amantadine is a synthetic tricyclic amine; it was initially used as an antiviral drug with specificity to inhibit replication of the Influenza A virus acting on the M2 protein, preventing viral uncoating and thus its replication (Aoki and Sitar, 1988). Its use as an antiviral drug is no longer recommended. Other uses of amantadine in clinical practice have been Parkinson’s disease, drug-induced extrapyramidal syndromes, multiple sclerosis, and catatonia. The most common adverse reactions reported in the literature are cardiovascular, such as orthostatic hypotension, presyncope, syncope, and peripheral edema, with an incidence of >10%. Neurological side effects such as dizziness, hallucinations, delusions, and paranoia are common too. Ataxia, confusion, fatigue, dyschromia, and suicidal ideation have been reported with less frequency (Babington and Spiegel, 2007; Pahwa, 2021).

Recently it has been noted that amantadine might have other significant mechanisms of action with direct and indirect effects on dopamine neurons such as inhibition of dopamine uptake and increased affinity of dopamine for D2 receptors, thereby increasing the release of dopamine, noradrenaline, and serotonin in the amygdala and hippocampus (de Lucena et al., 2012).

As previously described, catatonia might be due to hyper-excitability of PFC, SMA, thalamus, and limbic system, as well as an increased oxidative stress and neuroinflammation. Amantadine acts as a weak antagonist of NMDA receptors, and accelerates channel closure, thereby reducing excitotoxicity and hyper-excitability arising from calcium entry to the cell, and preventing mitochondrial stress. This diminishes RS production, avoiding cellular apoptosis and dysfunction (de Lucena et al., 2012; Fryml et al., 2017). Furthermore, by reducing excitotoxicity, it can in turn compensate GABA_A_ hypofunction at the mesostriatal circuit and reduce dopamine uptake at the synaptic cleft (Northoff et al., 1997; Roy et al., 2016).

Table 2 summarizes amantadine regimens used in catatonic patients and their clinical outcomes. Based on encouraging results reported in the literature, considering BDZs are not always effective, that amantadine has a longer time to treatment when compared to BDZs, and ECT is not widely available, we consider that clinical trials must be carried out to establish a therapeutic schedule to set amantadine as a first or common approach to patients with catatonia.

There are few reported cases where memantine was successfully used to treat catatonia, these cases were frequently associated with schizophrenia, also named catatonic schizophrenia (Thomas et al., 2005; Carpenter et al., 2006; Mukai et al., 2011; Obregon et al., 2011; Roy et al., 2016). Memantine is an antagonist of NMDAr, and it is a derivate from amantadine, therefore its mechanism of action is thought to be very similar to the one exposed earlier in this section. Memantine might be used when pro-dopaminergic effects of amantadine want to be avoided, but it must be carefully considered as its adverse effects include psychosis and seizures (Carpenter et al., 2006).

Minocycline is a tetracyclic-derived antibiotic that has come to our attention as there is evidence it reduces excitotoxicity and microglia activation, therefore attenuating neuroinflammation, OS, and apoptosis (Tikka et al., 2001). It is under study in many neurological pathologies (Cruz et al., 2020; Yang et al., 2020; Baracaldo-Santamaría et al., 2022). Regarding catatonia, Miyaoka et al. (2007) published a case series where minocycline successfully treated catatonia in concurrence with schizophrenia. As we stated earlier, microglia’s activation is a hallmark of catatonia. It was proven minocycline attenuates microglia’s reaction; thus, it is congruous to consider this antibiotic for the treatment of catatonia.

## Novel Therapies

### Repetitive transmagnetic stimulation

A case report published by Di Michele and Bolino described a patient diagnosed with bipolar type I disorder with a psychotic depressive episode and catatonia, and who was refractory to most common treatments (Di Michele and Bolino, 2006). Interestingly, an improvement in motor behavior was achieved by using Repetitive Transmagnetic Stimulation (rTMS; 10 sessions, 20 Hz) in the left dorsolateral prefrontal cortex with no other drugs administered. Encouraging evidence has triggered the application of rTMS in diverse psychiatric disorders with fruitful results (McClintock et al., 2018; Ocampo et al., 2022). rTMS is a non-invasive brain stimulation technique and aims to either increase or decrease cortical activity by applying different electrical frequency protocols (Begemann et al., 2020). When considered in catatonia, it might be useful as its mechanism of action, although not clearly elucidated, resembles that of ECT therapy, and might act by treating catatonia itself, or the underlying condition (Spampinato et al., 2013). Modulation of GABAergic transmission might be the mechanism of action of rTMS, and we speculate rTMS might work well when ECT is not available. Nonetheless, more research is needed to support its implementation (Trojak et al., 2014).

### Lithium therapy

A prospective study performed by Lee (1998) assessed lithium levels in catatonic patients, finding that a great proportion of these patients had low levels of lithium. In consequence, repletion therapy was started and, interestingly, its administration successfully treated catatonia. Similar cases are reported in the literature (Padhy et al., 2011; Sugawara et al., 2021). Even though there is no clear mechanism of action for lithium, it appears to stabilize the excitatory/inhibitory disbalance, that can be either generated by catatonia or the underlying disease beneath. It has been clearly stated that SMA hyperactivation and OFC/vmPFC hypofunction are common findings in catatonia, so reestablishing balance among circuitries is a clear solution to the disorder. Moreover, lithium has a protective role as it prompts homeostasis, and modulates OS and inflammatory cascades (Forlenza et al., 2014). Altogether, lithium is a promising therapy in catatonia as it acts in diverse pathological processes, as described earlier. Caution must be taken when prescribing lithium: important adverse effects are not common but when established they might have a great impact on the patients’ health (Thippaiah et al., 2021).

### Positive allosteric modulation of N-methyl-D-aspartate

Section “Amantadine and NMDAr antagonists” describes the mechanism of action of amantadine and other antagonists of NMDAr and its success in catatonia; additionally, Section “The role of neurotransmitters and circuitry dysfunction” demonstrates functional reconstitution of vmPFC mediated by NMDA blockers. In this section, we encourage considering NMDAR-positive allosteric modulators such as rapastinel and spermine to treat catatonia with psychoaffective predominance, as they can target both catatonia and psychiatric disease. Interestingly, rapastinel is a positive allosteric modulator whose mechanism of action resembles that of ketamine but without adverse effects such as cognitive impairment and psychomimetic symptoms (Kato and Duman, 2020). Rapastinel has shown encouraging results in patients with treatment-resistant major depressive disorder (Donello et al., 2019; Ragguett et al., 2019; Naurex, Inc, an affiliate of Allergan plc., 2020). By modulating NMDAR action, rapastinel could reverse hyperactivity of SMA, restore the inhibitory wave from basal ganglia and induce CaMK2A expression to improve NMDA-dependent synaptic plasticity. NMDA-positive allosteric modulators have a broad clinical application and have shown good safety and efficacy profile in many neurological disorders (Baracaldo-Santamaría et al., 2022; Geoffroy et al., 2022). Nonetheless, it is essential to consider that NMDA modulators have different targets, as the dynamic endogenous configuration of NMDAR is complex, and different brain areas express different NMDARs. We consider that rapastinel and spermine might have a positive impact in patients with catatonia (Velloso et al., 2009; Geoffroy et al., 2022).

### Cyclooxygenase-2 selective (COX-2) inhibitors—nonsteroidal anti-inflammatories

Taking into consideration that OS is a consequence of sustained metabolic stress and inflammation, while also bearing in mind the evidence provided by preclinical studies (Gupta et al., 2011), we consider COX-2 selective inhibitors might be regarded as a reasonable parallel therapeutic strategy to mitigate OS and inflammation, decreasing cellular damage and thus avoiding long-term consequences arising from catatonia. Even though there are no cases reported in the literature, rofecoxib and 1-(Phenyl)-5-(4-methylsulfonylphenyl)-2-ethylthioimidazole were used successfully in preclinical models (Gupta et al., 2009; Fathi-Moghaddam et al., 2010). The mechanisms by which COX-2 inhibitors might reduce catatonia vary from scavenger increase to enhancement of dopaminergic transmission at the striatum.

### Vitamin B12/cobalamin

B12 or Cobalamin is an essential water-soluble vitamin which plays an important role in maintaining physiological functions in the hematopoietic and central nervous systems; however, when deficient, B12 might cause demyelination of peripheral and central neurons (Green et al., 2017; Calderón-Ospina and Nava-Mesa, 2020). In a case reported by Bram et al. (2015), a 60-year-old woman diagnosed with Biermer’s disease was receiving a monthly IM dose of 1,000 μg/month of cyanocobalamin to treat her underlying condition. During admission, she started to present slow speech, apathy, minimal changes of facial expression, anxiety, a perplexed look, flat affect, poor spontaneous movements, waxy flexibility, and facial and manual stereotypies. Throughout time, consistently low levels of B12 were found, so finally the catatonic syndrome was resolved using 12 mg of lorazepam daily and cyanocobalamin injections every 3 weeks, the latter titrated to achieve B12 plasma concentrations of no less than 200 pg/ml. Few, but encouraging, cases report successful treatment of catatonia using cyanocobalamin injections (Berry et al., 2003). Whether deficiency of vitamin B12 is a cause, or a consequence of catatonia remains elusive; nonetheless, it is clear that vitamin B12 has an important antioxidant effect. The oxidative stress regulation mechanism encompasses scavenging of ROS, preservation of GSH, regulation of cytokine and growth factor production, reduction of homocysteine-induced OS, and diminishment of OS produced by advanced glycation end products (van de Lagemaat et al., 2019).

As it is well known, neither humans nor plants can produce cobalamin by themselves, said vitamin is exclusively obtained exogenously as a product of certain microorganisms, especially anaerobes. Different model organisms have been studied for the biosynthesis of cobalamin, among which are *P. freudenreichii*, an organism used in the commercial production of vitamin B12, and more recently *Lactobacillus reuteri* CRL1098. The latter lactic acid bacteria has shown to produce a compound which closely resembles cobalamin which has led investigators to believe the use of said bacteria during food production might be a natural way to increase vitamin B12 levels avoiding side effects coming from chemically synthesized products (Taranto et al., 2003; LeBlanc et al., 2013). As previously mentioned, vitamin B12 deficiency’s role in catatonia is yet to be further studied, however, as will be discussed in Section “Microbiota-gut-brain axis”, maintaining a healthy and appropriate gut microbiota, especially *Lactobacillus reuteri* CRL1098, might be a pivotal aspect in preventing catatonia and the inflammatory consequences that it entails.

### Microbiota-gut-brain axis

In recent decades, diverse studies have been carried out seeking to characterize and define the human microbiota. Today, microbiota are defined as a set of microorganisms that cohabit within humans (Lloyd-Price et al., 2016; Heintz-Buschart and Wilmes, 2018; Das and Nair, 2019). Microbiota contribute to body homeostasis through the regulation of metabolic processes, digestion and absorption of nutrients, regulation of gene expression, and modulation of proinflammatory and anti-inflammatory cytokines (Schirmer et al., 2016; Al Bander et al., 2020). Regarding CNS, the production of essential neurotransmitters and stabilization of BBB are worth noting as key functions of microbiota. Furthermore, a strong communication between the brain and gut has been proposed: the gut-brain axis. These systems are thought to be connected through the vagus nerve, and the lymphatic and circulatory systems. In this complex interaction, abnormalities at either end of the axis could therefore affect the other.

Considering the influence of microbiota on neurotransmitter production, and its modulation of BBB permeability and neuroinflammation, it is worth considering microbiota as a therapeutic target in CNS pathologies. Abundant literature describing the role of microbiota in autism spectrum disorders and SCZ, among others, has emerged (Jaskiw et al., 2019; Dong et al., 2022; Padhi et al., 2022). As described above, immune dysregulation is a hallmark in catatonia; in consequence, we consider that introducing prebiotics and probiotics could be promising as concurrent therapies in catatonia to help modulate BBB permeability, inflammation, and OS. An individualized characterization and posterior prescription of specific prebiotics and probiotics, or microbiota transfer therapy, can enhance microorganisms such as *F. prausnitzii, Acinetobacter spp., Bacteroides fragilis*, and *Proteobacteria*, which would stimulate short chain fatty acid production and in consequence could help to regulate systemic inflammation and therefore neuroinflammation (Blander et al., 2017). Nonetheless, as it is a new field of research, much speculation surrounds the literature, and careful assessment of each individual case must be carried out before initiating this therapy.

## Conclusions

In conclusion, this review has described and assessed the broad literature surrounding catatonia and similar conditions to frame a translational explanation of the mechanisms underlying catatonia’s etiology. The mechanisms underlying the onset of this syndrome are complex and involve variable aspects including the patient’s condition and comorbidities. A relatively high frequency of catatonic syndrome is observed in patients with psychiatric conditions, with mood disorders being the most frequent, followed by psychotic syndrome and acute illness. Available evidence from clinical, neuroimaging, and preclinical research encourage us to list catatonia’s hallmarks as inhibitory/excitatory imbalance, neuroinflammation, and OS. Additionally, the interactions between astrocytes, neurons, oligodendrocytes, and microglia determine the onset and progression of catatonia. Nonetheless, what exactly triggers catatonic symptoms is yet to be elucidated. When it comes to treatment, evidence suggests clinicians should always aim to treat the underlying condition first as it has been proven to be the most effective therapy. Current therapeutics such as BDZ and ECT are usually enough to resolve the syndrome when available, while other drugs such as amantadine and lithium are available and have shown consistent results to treat this condition. Bearing in mind the molecular and cellular mechanisms described and the lack of evidence around them, we encourage further research of other treatments like vitamin B12, COX inhibitors, microbiota reestablishment, and positive allosteric modulation of NMDAR to control OS and inflammation, as well as to reestablish the excitatory/inhibitory balance. Finally, encouraging research has been made on this disorder, nonetheless, there is still much more research to be done to fill the remaining voids.

## Author Contributions

DA-S: conceptualization, writing—review and editing, and Figure 2 construction. DA-S, MC-H, and MP-L: writing—original draft preparation. MC-H and DA-S: Figure 1 construction. IH-D: Table 1 construction. MP-L: Table 2 construction. All authors contributed to the article and approved the submitted version.

## Funding

This project was supported by the Universidad del Rosario.

## Figures and Tables

**Table:** 

NMDAr	N-methyl-D-Aspartate Receptor
SMA	Supplementary Motor Area
OFC	Orbitofrontal Cortex
PFc	Prefrontal Cortex
GABA-A	Gamma-Aminobutyric acid-A
NMDA	N-Methyl-D-Aspartate
CN	Caudate Nucleus
VS	Putamen and Ventral Striatum
iGP	Internal Globus Pallidus
eGP	ExternalGlobus Pallidus
SNc	Substantia Nigra Pars Compacta
SNr	Substantia Nigra Pars Reticulata
STN	Subthalamic Nucleus
VL	Ventral Lateral Nuclei of the Thalamus
VA	Ventral Anterior Nuclei of theThalamus
D1R	Dopamine Type 1 Receptor
D2R	Dopamine Type 2 Receptor
SCZ	Schizophrenia
vmPFC	Ventromedial Prefrontal Cortex
SPG	Superior Left Parietal Gyrus
SMG	Supramarginal Gyrus
MPF	Medial Prefrontal Cortex
CAC	Cingulate Anterior Cortex
NIC	Neuroleptic-Induced Catatonia
TNF alpha	Tumour Necrosis Factoralpha
NO	Nitric Oxide
Cnp	2’,3’- cyclic nucleotide 3’-phosphodiesterase
OS	Oxidative Stress
RS	Reactive Species
ROS	Reactive Oxygen Species
RNS	Reactive Nitrogen Species
H_2_O_2_	HydrogenPeroxide
ROO	Peroxyl Radicals
O_2_^−^O_2_^−^	Superoxide Anion Radical
OH	Hydroxyl Radical
ONOO-	Peroxynitrite
O_2_^−^	Single Molecular Oxygen
SOD	Superoxide Dismutase
GSH	Glutathione
RAGE	Receptor for Advanced Glycation End-Products
Nf-KB	Nuclear factor kappa-light-chain-enhancer ofactivated B cells
BBB	Blood-Brain Barrier
BZD	Benzodiazepines
ECT	Electroconvulsive Therapy
IM	Intramuscular
IST	Initial Seizure Threshold
rTMS	Repetitive Transmagnetic Stimulation
NMS	Neuroleptic Malignant Syndrome
CNS	Central Nervous System
